# The Acute Effects of Swimming Exercise on PGC-1α-FNDC5/Irisin-UCP1 Expression in Male C57BL/6J Mice

**DOI:** 10.3390/metabo11020111

**Published:** 2021-02-16

**Authors:** Eunhee Cho, Da Yeon Jeong, Jae Geun Kim, Sewon Lee

**Affiliations:** 1 Department of Human Movement Science, Graduate School, Incheon National University, Incheon 22012, Korea; j_hee1009@naver.com; 2 Division of Life Sciences, College of Life Sciences and Bioengineering, Incheon National University, Incheon 22012, Korea; dayeon@inu.ac.kr (D.Y.J.); jgkim@inu.ac.kr (J.G.K.); 3 Institute for New Drug Development, Division of Life Sciences, Incheon National University, Incheon 22012, Korea; 4 Division of Sport Science, College of Arts & Physical Education, Incheon National University, Incheon 22012, Korea; 5 Sport Science Institute, College of Arts & Physical Education, Incheon National University, Incheon 22012, Korea; 6 Health Promotion Center, College of Arts & Physical Education, Incheon National University, Incheon 22012, Korea

**Keywords:** irisin, swimming exercise, UCP1, PGC-1α, FNDC5

## Abstract

Irisin is a myokine primarily secreted by skeletal muscles and is known as an exercise-induced hormone. The purpose of this study was to determine whether the PGC-1α -FNDC5 /Irisin-UCP1 expression which is an irisin-related signaling pathway, is activated by an acute swimming exercise. Fourteen to sixteen weeks old male C57BL/6J mice (*n* = 20) were divided into control (CON, *n* = 10) and swimming exercise groups (SEG, *n* = 10). The SEG mice performed 90 min of acute swimming exercise, while control (non-exercised) mice were exposed to shallow water (2 cm of depth) for 90 min. The mRNA and protein expression of PGC-1α, FNDC5 and browning markers including UCP1 were evaluated by quantitative real-time PCR and western blotting. Serum irisin concentration was measured by enzyme-linked immunosorbent assay. An acute swimming exercise did not lead to alterations in the mRNA and protein expression of PGC-1α in both soleus and gastrocnemius muscles, the mRNA and protein expression of UCP1 in brown adipose tissue, mRNA browning markers in visceral adipose tissue and circulating irisin when compared with the control group. On the other hand, an acute swimming exercise led to increases in the mRNA and protein expressions of FNDC5 in the soleus muscle, the protein expression of FNDC5 in the gastrocnemius muscles and the protein expression of UCP1 in subcutaneous adipose tissue.

## 1. Introduction

Irisin is a myokine secreted primarily by skeletal muscles and is known as an exercise-induced hormone [1]. Exercise-induced muscle contraction stimulates the gene expression for fibronectin type III domain-containing protein 5 (FNDC5), and irisin is cleaved from FNDC5 and released in circulation [1]. FNDC5 is controlled by peroxisome proliferator-activated receptor-gamma coactivator-1α (PGC-1α) in response to chronic exercise and is positively related to PGC-1α in human skeletal muscle [2].

PGC-1α and FNDC5 expression and increment in circulating level of irisin are known to enhance energy consumption and stimulate the browning of white adipose tissue (WAT), thereby ameliorating insulin resistance [3]. In addition, recent studies have reported that irisin had a protective effect on the dysfunction of pancreatic β cell [4,5] and vascular dysfunction [6]. In this regard, irisin is attracting attention as a signaling mechanism that can be used to treat metabolic diseases such as obesity, insulin resistance, and metabolic syndrome because it increases the expression of uncoupling protein 1 (UCP1) present in the inner membrane of mitochondria, thereby inducing the browning of WAT. 

It is well known that aerobic exercise improves metabolic health including weight management, glucose and cholesterol metabolism for the prevention and treatment of metabolic abnormalities [7]. In particular, increased blood levels of irisin with regular exercise improved energy metabolism and glucose homeostasis, suggesting that it may improve obesity, insulin resistance and its complications [8]. 

However, the effects of exercise on irisin and irisin-related signaling substances expression are still under discussion and controversial because different results were reported depending on the type of exercise (swimming vs. running, differences of recruited muscles), intensity, frequency and duration. Previous studies indicated that the circulating level of irisin was increased with acute endurance exercise in experimental animal models and humans [9,10,11]. Increased level of irisin is suggested to mediate the alteration of WAT browning by increasing energy consumption through the UCP1 expression, and it is evident that browning of WAT is related to the beneficial effect of exercise [1,12,13,14,15]. On the other hand, other studies suggested no effect of acute endurance exercise on circulating irisin [16,17]. Interestingly, Tsuchiya et al. indicated an acute bout of resistance exercise caused greater irisin responses compared with endurance exercise alone or combined resistance and endurance exercise [16]. Although previous studies reported that exercise activates those factors, studies on whether acute swimming exercise promotes this signaling pathway are still unclear. In addition, it is also uncertain whether the activation patterns appear differently according to muscle fiber types (Type I vs. Type II) and fat types (brown adipose tissue (BAT) vs. white adipose tissue). Therefore, the purpose of this study was to determine whether the PGC-1α-FNDC5/Irisin-UCP1 expression, which is an irisin-related signaling pathway, is activated by an acute swimming exercise.

## 2. Results 

### 2.1. An Acute Swimming Exercise Did Not Increase mRNA and Protein Expressions of PGC-1 α in 2 Different Types of Skeletal Muscle Fibers

**Figure 1:**
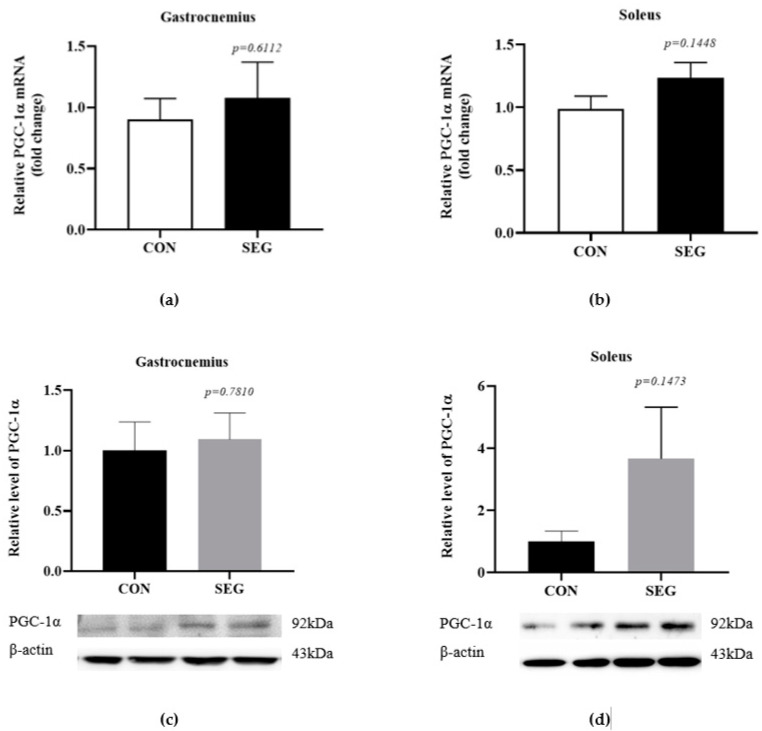
An acute swimming exercise did not increase mRNA and protein expressions of PGC-1α in skeletal muscles. Male C57BL/6J mice were exposed to an acute swimming exercise for 90 min (SEG) or to shallow water for 90 min (CON). The levels of *PGC-1α* mRNA were comparable between groups in gastrocnemius (**a**) and soleus (**b**) muscles. In addition, the levels of PGC-1α protein were also comparable between groups in gastrocnemius (**c**) and soleus (**d**) muscles. Results are presented as mean ± SEM. *n* = 6–10 mice per group. CON, control group, SEG; swimming exercise group.

A previous report suggested that chronic voluntary wheel running exercise activated PGC-1α expression in skeletal muscle, which promoted FNDC5 expression in skeletal muscle and irisin concentration in circulation [1]. In order to verify whether a 90 min acute swimming exercise activates PGC-1α in skeletal muscles, we evaluated mRNA and protein expression in gastrocnemius and soleus muscles which were mixed muscle fiber (fast and slow) and slow-twitch muscle fiber, respectively, between control (CON) and swimming exercise group (SEG). The mRNA expression of *PGC-1α* was comparable between groups in gastrocnemius (Figure 1a) and soleus (Figure 1b) muscles. The protein expression of PGC-1α was also examined in both soleus and gastrocnemius muscles in CON and SEG and the levels of PGC-1α protein were also comparable between groups in gastrocnemius (Figure 1c) and soleus (Figure 1d) muscles. There was a tendency toward an increase in the mRNA and protein expressions of PGC-1α in soleus muscle but it did not reach to the statistical difference (*p* = 0.1448, *p* = 0.1473, respectively, Figure 1b,d). 

### 2.2. An Acute Swimming Exercise Activates mRNA and Protein Expressions of FNCD5 in 2 Different Types of Skeletal Muscle Fibers 

**Figure 2:**
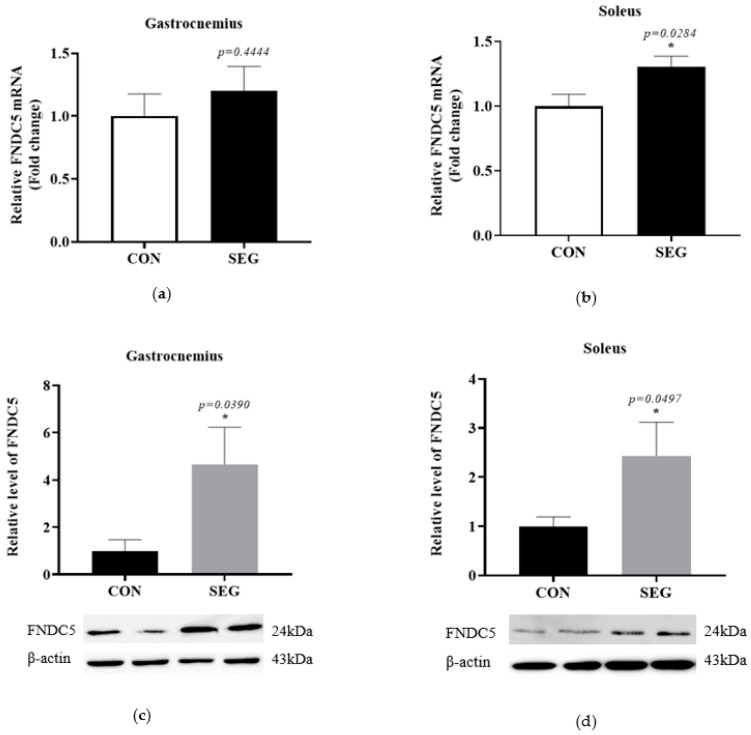
An acute swimming exercise increased mRNA and protein expressions of FNDC5 in skeletal muscles. Male C57BL/6J mice were exposed to an acute swimming exercise for 90 min (SEG) or to shallow water for 90 min (CON). The level of *FNDC5* mRNA was comparable between groups in gastrocnemius (**a**), whereas the level of that was significantly increased in SEG compared to CON in soleus muscle (**b**). In addition, the levels of FNDC5 protein were also significantly increased in SEG compared to CON in both gastrocnemius (**c**) and soleus muscles (**d**). Results are presented as mean ± SEM. *n* = 7–10 mice per group. * *p* < 0.05 vs. CON. CON, control group; SEG, swimming exercise group.

To determine whether an acute swimming exercise activates FNDC5 in skeletal muscles, we identified mRNA and protein expression of FNDC5 in two different types of skeletal muscle fibers. The mRNA expression of *FNDC5* was comparable between groups in gastrocnemius (*p* = 0.4444, Figure 2a), while the mRNA *FNDC5* was significantly increased in SEG compared to CON in soleus (*p* = 0.0497, Figure 2b) muscle. The protein expressions of FNDC5 were also examined in both soleus and gastrocnemius muscles in CON and SEG. The levels of FNDC5 protein were significantly increased after the acute exercise in both gastrocnemius (4.657 folds increase, Figure 2c) and soleus (2.435 folds increase, Figure 2d) muscles. 

### 2.3. An Acute Swimming Exercise Did Not Increase Circulating Irisin in Serum

**Figure 3:**
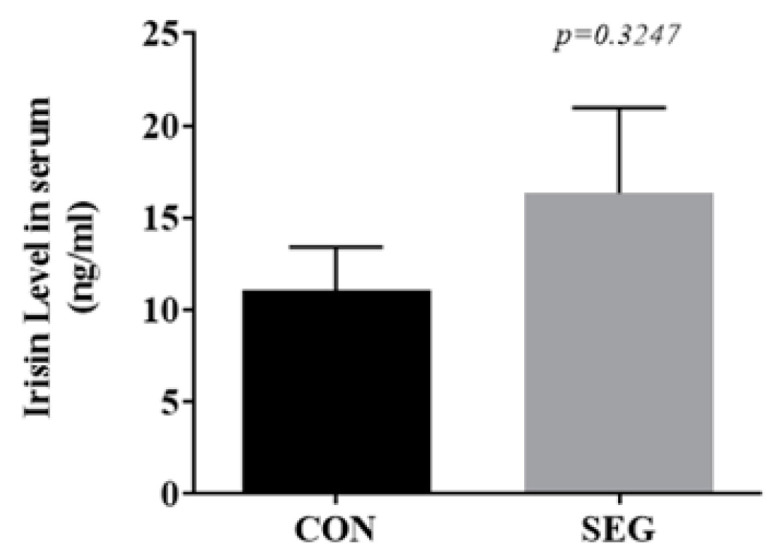
An acute swimming exercise did not increase circulating irisin concentration in serum. Male C57BL/6J mice were exposed to an acute swimming exercise for 90 min (SEG) or to shallow water for 90 min (CON). Results are presented as mean ± SEM. *n* = 9 mice per group. CON, control group; SEG, swimming exercise group.

The activation of FNDC5 in skeletal muscles resulted in an elevation in the level of circulating irisin which causes the browning of WAT by UCP1 [18]. Therefore, to verify the acute effect of swimming exercise on circulating irisin, we used an enzyme-linked immunosorbent assay (ELISA) with an antibody specific to irisin. An acute swimming exercise for 90 min did not lead to an increase in circulating irisin (*p* = 0.3247, Figure 3). 

### 2.4. An Acute Swimming Exercise Increases Protein Expression of UCP1 in Subcutenous White Adipose Tissue 

**Figure 4:**
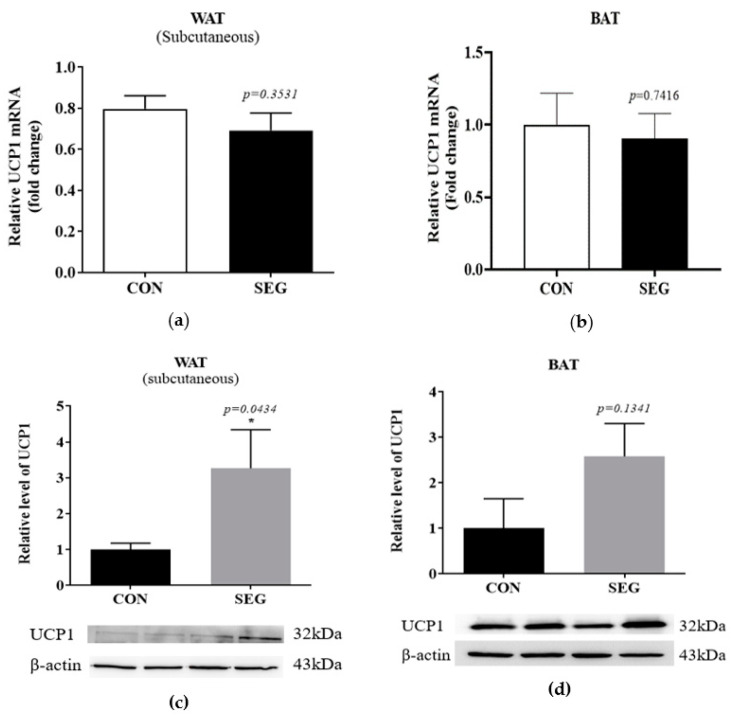
An acute swimming exercise increase UCP1 protein expression in subcutaneous WAT, whereas it did not change *UCP1* mRNA expression in subcutaneous WAT and BAT and UCP1 protein expression in BAT. Male C57BL/6J mice were exposed to an acute swimming exercise for 90 min (SEG) or to shallow water for 90 min (CON). The level of *UCP1* mRNA was comparable between groups in both subcutaneous WAT (**a**) and BAT (**b**). In addition, the level of UCP1 protein was significantly increased in SEG compared to CON in subcutaneous WAT (**c**), whereas the level of UCP1 protein was comparable in BAT (**d**) between groups. Results are presented as mean ± SEM. *n* = 5–9 mice per group. * *p* < 0.05 vs. CON. BAT, brown adipose tissue; CON, control group; SEG, swimming exercise group; WAT, white adipose tissue.

To test whether an acute swimming exercise increased UCP1 in adipose tissues, we examined mRNA and protein expression in 2 different types of adipocytes which were subcutaneous adipocytes and brown adipocytes. The *UCP1* mRNA in subcutaneous WAT and BAT after an acute swimming exercise was not different from that of CON (Figure 4a,b), whereas the protein expression of UCP1 was significantly increased in SEG compared to CON (Figure 4c). The protein expression in BAT did not alter by an acute swimming exercise (*p* = 0.1341, Figure 4d).

### 2.5. Change in mRNA Expression of Browning Markers in Visceral WAT after Acute Swimming Exercise 

**Figure 5:**
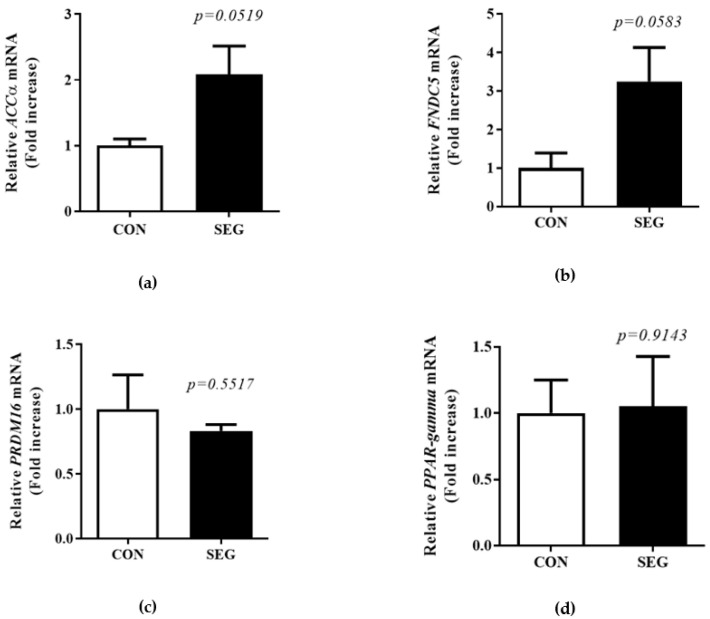
Change in browning markers mRNA expression in visceral WAT after acute swimming exercise. Male C57BL/6J mice were exposed to an acute swimming exercise for 90 min (SEG) or to shallow water for 90 min (CON). The level of *ACCα* mRNA (**a**), and *FNDC5* mRNA (**b**) was not significantly increased in SEG compared to CON in visceral WAT. The mRNA expression of *PRDM16* (**c**) and *PPARγ* mRNA (**d**) was comparable between groups. Results are presented as mean ± SEM. *n* = 4 mice per group. * *p* < 0.05 vs. CON. CON, control group; SEG, swimming exercise group; WAT, white adipose tissue.

A previous study showed acute treadmill exercise did not change mRNA *UPC1* expression in visceral WAT [19]. To test whether an acute swimming exercise increased browning in visceral WAT, we examined browning marker mRNA in visceral WAT. There was a tendency toward an increase in the mRNA of acetyl-CoA carboxylase (*ACC*)α and *FNDC5* in visceral WAT, but it did not reach to the statistical difference (*p* = 0.0519, *p* = 0.0583, respectively. Figure 5a,b). In addition, the mRNA expression of *PRDM16* and peroxisome-proliferator-activated receptor-gamma (*PPARγ*) in visceral WAT did not alter by an acute swimming exercise (*p* = 0.5517, *p* = 0.9142, respectively. Figure 5c,d).

## 3. Discussion

The purpose of this study was to determine whether the PGC-1α-FNDC5/Irisin-UCP1 expression, which is an irisin-related signaling pathway, is activated by an acute swimming exercise. One previous study found that when mice were treated with voluntary wheel running exercise for three weeks, the expressions of *PGC-1α* and *FNDC5* genes were increased in skeletal muscles, and the expression of the *UCP1* gene was increased in adipose tissues [1]. However, studies on whether even acute swimming exercise activates the PGC-1α-FNDC5 /Irisin-UCP1 signaling mechanism and whether the activation patterns appear differently according to muscle fiber types (Type I vs. Type I and II mixed) and fat types (brown fat vs. white fat) are insufficient. Our hypothesis was that irisin-related signaling substances would increase after 90 min of acute swimming exercise. However, unlike the expectation, there were no differences in the mRNA and protein expressions of PGC-1α in skeletal muscles, the mRNA and protein expression of UCP1 in brown adipose tissue, circulating irisin and browning markers in visceral WAT in the acute swimming group when compared with the control group. On the other hand, an acute swimming exercise led to increases in the expression of FNDC5 protein in both the soleus muscle (2–3 folds increase) that has many type I muscle fibers and the gastrocnemius muscle (4–5 folds increase) that has both type I and II muscle fibers. In this study, only the expression of FNDC5 in skeletal muscle increased, while the expression of PGC-1α was not increased after the exercise. In a previous study conducted with human, alteration of mRNA *PGC-1α* expression in skeletal muscle or serum irisin was not accompanied by corresponding changes in FNDC5 [20]. Moreover, they suggested that large intra- and inter-individual variations in *PGC-1α, FNDC5* gene expression and circulating irisin in response to a single resistance exercise and heavy-intensity endurance training [20]. In addition, a mouse study conducted by Pang et al. has reported that PGC-1α expression increased during 30 min of treadmill exercise but decreased during 1 h of treadmill exercise, suggesting that the regulation of gene and protein expression may differ according to the duration of exercise [11]. A few studies suggested 6-h prolonged acute bout of swimming exercise-induced an elevated mRNA expression of *PGC-1* immediately after [21] and elevation of PGC-1α protein was maintained 18 and 24 h after the exercise in rat epitrochlearis muscle [22]. Given these previous findings, the expression of PGC-1α is considered to occur when some time has passed after exercise. Therefore, the duration of exercise is considered to play an important role in the regulation of the PGC-1α-FNDC5/Irisin-UCP1 signaling mechanism. Furthermore, the changes in FNDC5 expression were not accompanied by changes in PGC-1α, suggesting that a factor other than PGC-1α may be involved in the regulation of FNDC5 expression. Although FNDC5 is known as PGC-1α dependent myokine, the expression of FNDC5 increased without PGC-1α activation in our study. Indeed, the increase in FNDC5 expression is not necessarily caused by PGC-1α activation and further study is needed on the irisin-related mechanisms involved in FNDC5 expression. For example, Natalicchio et al. shown that higher irisin concentration by palmitate occurred without an increase in *FNDC5* mRNA levels in human myotubes [5]. Furthermore, in our study the increases in FNDC5 expression were not accompanied by an increase in serum irisin, indicating that different signaling processes may be involved in irisin release from other tissues in addition to transcriptional regulation in skeletal muscles. For instance, Roca-Rivada et al. shown that FNDC5 protein was produced and released from both visceral and subcutaneous adipose tissue in a rat model [23]. Thus, it seems that various upstream mechanisms induced by exercise can affect the expression of PGC-1α-FNDC5/Irisin-UCP1 expression.

The results of previous studies that examined the irisin response after exercise are controversial. Contrary to previous studies which showed increased circulating irisin concentration after exercise [2,24,25], inconsistent studies were also reported [2,20,26]. A previous study showed prolonged, moderate-intensity aerobic exercise increased circulating irisin concentration at 54 min and then decreased at 90 min during an acute treadmill exercise [27]. In the previous study, circulating irisin level was further declined by 20 min of the recovery period in humans [27]. In addition, Nygaard et al. have reported that the concentration of irisin was transiently increased after aerobic exercise and then decreased over time and returned to the pre-exercise concentration after 6 h [9]. These two previous studies have indicated that acute exercise may lead to a transient increase in irisin and the elevated irisin concentration was maintained for a short time.

In the present study, to create an environment similar to the swimming exercise group, the control group mice were exposed to shallow water for 90 min in a water tank filled with water to approximately 2 cm heights. Although we did not measure the body temperature of CON mice, it is possible that the body temperature of the CON mice might be declined during this period of time and led to increases in the irisin-related signaling substances such as PGC-1α and irisin in the control group. For this reason, we may not see the differences between CON and SEG in PGC-1α and irisin. As a precedent, it was found that in the white adipose of rat exposed to cold, the level of UCP expression almost reached the level of mitochondria presented in brown fat [28] and Lee et al. also suggest that exercise-induced irisin secretion could have evolved from shivering-related muscle contraction to increase brown fat thermogenesis [29], indicating cold-induced thermogenesis may affect the irisin-related signaling pathway. More detailed studies are required to further investigate the effect of shallow water exposure on the irisin-related signaling pathway. In addition, swimming exercise may be different from treadmill exercise or voluntary wheel running exercise because it leads to a change in body temperature during exercise, which results in an alteration in the irisin-related signaling pathway. In addition, a study suggested that 6-h prolonged swimming exercise reduced muscle glycogen concentrations in the epitrochlearis, triceps and red region of the gastrocnemius muscles, whereas it did not change in soleus, plantaris and white region of the gastrocnemius muscle in the experimental rat model [30]. On the other hand, the 6-h prolonged treadmill running caused a reduction in glycogen concentration in soleus, plantaris and red region of gastrocnemius muscle [30]. Furthermore, the protein expression of PGC-1α was significantly elevated by treadmill exercise, not a swimming exercise in rat soleus muscle, suggesting different parts of muscles may be stimulated according to exercise type (treadmill vs. swimming) [30]. In this study, we did not separate the white and red regions of the gastrocnemius muscle. Because the red region of the gastrocnemius muscle was highly recruited both by swimming and running exercise, it is necessary to investigate the signaling pathways using the red part of the gastrocnemius muscle in the future. Therefore, well-controlled additional studies are needed to verify whether acute or chronic swimming exercises activate the PGC-1α-FNDC5/Irisin-UCP1 signaling pathway.

## 4. Materials and Methods 

### 4.1. Experimental Animal Model

Fourteen to sixteen weeks old male C57BL/6J mice (*n* = 20) were purchased from the Shizuoka Laboratory Center (Shizuoka, Japan). The mice were housed on a 12-h light/dark cycle (7:00 a.m., lights on and 7:00 p.m., lights off) and were given access to a standard chow diet and water ad libitum. The animal facility was controlled under specific pathogen-free conditions at 22 °C. All of the animal care and lab experimental procedures were performed in accordance with the Animal Care and Use Committee of the Incheon National University (Incheon, South Korea, permission# INU-ANIM-2018-17).

### 4.2. Procedure for an Acute Swimming Exercise

Before control and exercise treatment, all mice were acclimatized to swim in a glass water tank for 5 min/day for a week. After acclimation for swimming exercise, control and swimming exercise mice were randomly assigned to control (CON, *n* = 10) and swimming exercise group (SEG, *n* = 10). The SEG mice performed 90 min of acute swimming exercise in an environment of more than 15 cm deep in a glass water tank (45 × 45 × 45 cm), and 31 ± 1 °C water temperature according to protocols based on the previous studies [31,32]. Control (non-exercised) mice were exposed to shallow water (2 cm of depth) for 90 min. The CON mice could stand normally with their head out of the water and did not have to swim. After exercise and control treatment, all mice were gently dried with a cloth towel and then immediately anesthetized.

### 4.3. Preparations of Serum and Tissues

Following the completion of the treatment, all mice were anesthetized by an intraperitoneal injection of 2.5% tribromoethanol (0.01 mL/g of body weight). Under anesthesia, whole blood was obtained from the vena cava and held for 30 min at room temperature. Then, the blood sample was centrifuged at 12,000 rpm at 4 °C for 10 min and sera were transferred in separate tubes without disturbing blood clots. After that, soleus and gastrocnemius muscles, anterior subcutaneous WAT, epididymal WAT and interscapular BAT from mouse were rapidly excised respectively and transferred in separate tubes. All collected samples were stored at −80 °C refrigerator until the analysis.

### 4.4. Quantitative Real-Time PCR

Total RNA was isolated from the skeletal muscles, BAT, subcutaneous and visceral WAT, and reverse-transcribed to obtain cDNA using a Maxime RT PreMix kit (Intron Biotechnology, Seoul, South Korea). Real-time PCR amplification of the cDNA was analyzed with SYBR Green Real-time PCR Master Mix (Toyobo Co. Ltd., Osaka, Japan) in a Bio-Rad CFX 96 Real-Time Detection System (Bio-Rad Laboratories, Hercules, CA, USA). The results were analyzed using the CFX Manager software and normalized by a housekeeping gene, *β-actin*. The primers used were as follows: For *β-actin*: forward: 5′-TAA AAC GCA GCT CAG TAA CAG TCC G-3′ Reverse: 5′-TGG AAT CCT GTG GCA TCC ATG AAA C-3′. For *FNDC5*: forward: 5′AGA AGA AGG ATG TGC GGA TG-3′ reverse: 5′-TCT TGA AGA GCA CAG GCT CA-3′. For *PGC-1α*: forward: 5′-AAT GCA GCG GTC TTA GCA CT-3′ reverse: 5′-GTG TGA GGA GGG TCA TCG TT3′. For *UCP-1*: forward: 5′-GCG TTC TGG GTA CCA TCC TA-3′ reverse: 5′-GCT CTG AGC CCT TGG TGT AG-3′. For *ACCα*: forward: 5′-GAA GTC AGA GCC ACG GCA CA-3′ reverse: 5′-GGC AAT CTC AGT TCA AGC CAG TC-3′. For *PRDM16*: forward: 5′-AGC ACG GTG AAG CCA TTC-3′ reverse: 5′- GCG TGC ATC CGC TTG TG-3′. For *PPAR-γ*: 5′-TGT CGG TTT CAG AAG TGC CTT G-3′ reverse: 5′-TTC AGC TGG TCG ATA TCA CTG GAG-3′.

### 4.5. Procedure for Western Blotting

In this experiment, a conventional western blot system (Bio-Rad Laboratories, Hercules, CA, USA). Proteins in skeletal muscles and adipose tissues were analyzed by electrophoresis USA) was used to analyze specific protein levels using a typical type of electrophoresis. The skeletal muscles and adipose tissues collected were homogenized with CelLytic MT lysis buffer (Sigma-Aldrich, St Louis, MO, USA) mixed with protease inhibitors cocktail (Sigma-Aldrich, St Louis, MO, USA). The total protein concentration was measured by a BCA protein assay kit (Thermo Fisher Scientific, Rockford, IL, USA). An equal amount of proteins was separated by electrophoresis using sodium dodecyl sulphate (SDS)-polyacrylamide gel and then transferred to PVDF membrane. After the membranes were blocked with 5% skim milk, the proteins were incubated with the use of primary antibodies at the given dilutions: β-actin (Santa Cruz Biotechnology, catalog# sc-47778, 1:1000), PGC-1α, (Abcam, catalog# ab-54481, 1:500), FNDC5 (Abcam, catalog# ab-174833, 1:500) and UCP1 (Abcam, catalog# ab-10983, 1:1000). After incubation, wash steps were performed using TBST mixed with tris-buffered saline (TBS) and tween 20 twice for 5 min and twice for 10 min. The membranes were incubated with a secondary antibody (Abcam, 1:2000) for 1 h at room temperature. Thereafter, the wash procedure was performed twice for 5 min and 10 min twice for 30 min. The final band intensity was quantified using a Chemidoc Touch Imaging System (Bio-Rad Laboratories, Hercules, CA, USA) and normalized to that of the corresponding internal reference, β-actin.

### 4.6. Procedure for ELISA

Serum irisin concentration was measured according to the manufacturer’s experimental procedure using a commercial ELISA kit (AdipoGen Life Sciences, San Diego, CA, USA, AG-45A-0046YEK-KI01). To produce a standard curve of optical density (OD) versus irisin concentration, we added specimens, standard samples and HRP-labeled antibodies to micro-pores pre-coated with the irisin antibody, and the OD values of the standard samples and specimens were then detected with a microplate spectrophotometer at a wavelength of 450 nm. The concentration of irisin in the samples was subsequently determined by comparing the OD value of the samples to the standard curve.

### 4.7. Statistical Analysis

All values were presented as means ± SEM. All statistical data analysis was performed using GraphPad Prism 6.05 version software (La Jolla, CA, USA). The means of independent two groups in mRNA and protein expression and circulating irisin concentration were assessed using unpaired *t*-tests. The significance level was set as *p* < 0.05.

## 5. Conclusions

An acute swimming exercise did not lead to elevations in the mRNA and protein expressions of PGC-1α in skeletal muscles, the mRNA and protein expression of UCP1 in BAT, circulating irisin and browning markers in visceral WAT when compared with the control group. On the other hand, an acute swimming exercise led to increases in the mRNA and protein expressions of FNDC5 in the soleus muscle, the protein expression of FNDC5 in the gastrocnemius muscles and the protein expression of UCP1 in subcutaneous WAT. Our results demonstrated that an acute swimming exercise for 90 min appears to activate the parts of PGC-1α-FNDC5/Irisin-UCP1 signaling components.

## Data Availability

The data presented in this study are available in article.

## References

[1] Bostrom P., Wu J., Jedrychowski M.P., Korde A., Ye L., Lo J.C., Rasbach K.A., Bostrom E.A., Choi J.H., Long J.Z. (2012). A PGC1-alpha-dependent myokine that drives brown-fat-like development of white fat and thermogenesis. Nature.

[2] Norheim F., Langleite T.M., Hjorth M., Holen T., Kielland A., Stadheim H.K., Gulseth H.L., Birkeland K.I., Jensen J., Drevon C.A. (2014). The effects of acute and chronic exercise on PGC-1alpha, irisin and browning of subcutaneous adipose tissue in humans. FEBS J..

[3] Zhang Y., Li R., Meng Y., Li S., Donelan W., Zhao Y., Qi L., Zhang M., Wang X., Cui T. (2014). Irisin stimulates browning of white adipocytes through mitogen-activated protein kinase p38 MAP kinase and ERK MAP kinase signaling. Diabetes.

[4] Zhang D., Xie T., Leung P.S. (2018). Irisin Ameliorates Glucolipotoxicity-Associated beta-Cell Dysfunction and Apoptosis via AMPK Signaling and Anti-Inflammatory Actions. Cell Physiol. Biochem..

[5] Natalicchio A., Marrano N., Biondi G., Spagnuolo R., Labarbuta R., Porreca I., Cignarelli A., Bugliani M., Marchetti P., Perrini S. (2017). The Myokine Irisin Is Released in Response to Saturated Fatty Acids and Promotes Pancreatic beta-Cell Survival and Insulin Secretion. Diabetes.

[6] Byun K., Lee S. (2020). The Potential Role of Irisin in Vascular Function and Atherosclerosis: A Review. Int. J. Mol. Sci..

[7] Wu J., Bostrom P., Sparks L.M., Ye L., Choi J.H., Giang A.H., Khandekar M., Virtanen K.A., Nuutila P., Schaart G. (2012). Beige adipocytes are a distinct type of thermogenic fat cell in mouse and human. Cell.

[8] Perakakis N., Triantafyllou G.A., Fernandez-Real J.M., Huh J.Y., Park K.H., Seufert J., Mantzoros C.S. (2017). Physiology and role of irisin in glucose homeostasis. Nat. Rev. Endocrinol..

[9] Nygaard H., Slettalokken G., Vegge G., Hollan I., Whist J.E., Strand T., Ronnestad B.R., Ellefsen S. (2015). Irisin in blood increases transiently after single sessions of intense endurance exercise and heavy strength training. PLoS ONE.

[10] Aydin S., Kuloglu T., Aydin S., Eren M.N., Celik A., Yilmaz M., Kalayci M., Sahin I., Gungor O., Gurel A. (2014). Cardiac, skeletal muscle and serum irisin responses to with or without water exercise in young and old male rats: Cardiac muscle produces more irisin than skeletal muscle. Peptides.

[11] Pang M., Yang J., Rao J., Wang H., Zhang J., Wang S., Chen X., Dong X. (2018). Time-Dependent Changes in Increased Levels of Plasma Irisin and Muscle PGC-1alpha and FNDC5 after Exercise in Mice. Tohoku J. Exp. Med..

[12] Wu M.V., Bikopoulos G., Hung S., Ceddia R.B. (2014). Thermogenic capacity is antagonistically regulated in classical brown and white subcutaneous fat depots by high fat diet and endurance training in rats: Impact on whole-body energy expenditure. J. Biol. Chem..

[13] Sutherland L.N., Bomhof M.R., Capozzi L.C., Basaraba S.A., Wright D.C. (2009). Exercise and adrenaline increase PGC-1{alpha} mRNA expression in rat adipose tissue. J. Physiol..

[14] Brenmoehl J., Albrecht E., Komolka K., Schering L., Langhammer M., Hoeflich A., Maak S. (2014). Irisin is elevated in skeletal muscle and serum of mice immediately after acute exercise. Int. J. Biol. Sci..

[15] Stanford K.I., Middelbeek R.J., Goodyear L.J. (2015). Exercise Effects on White Adipose Tissue: Beiging and Metabolic Adaptations. Diabetes.

[16] Tsuchiya Y., Ando D., Takamatsu K., Goto K. (2015). Resistance exercise induces a greater irisin response than endurance exercise. Metabolism.

[17] Czarkowska-Paczek B., Zendzian-Piotrowska M., Gala K., Sobol M., Paczek L. (2014). One session of exercise or endurance training does not influence serum levels of irisin in rats. J. Physiol. Pharmacol..

[18] Xiong X.Q., Chen D., Sun H.J., Ding L., Wang J.J., Chen Q., Li Y.H., Zhou Y.B., Han Y., Zhang F. (2015). FNDC5 overexpression and irisin ameliorate glucose/lipid metabolic derangements and enhance lipolysis in obesity. Biochim. Biophys. Acta.

[19] Namgoong H., Lee J.-S., Kim J.-G., Lee S. (2018). Acute Effects of Aerobic Treadmill Exercise Intensity on Expression of Irisin and FNDC5 in Male Mouse. Exerc. Sci..

[20] Pekkala S., Wiklund P.K., Hulmi J.J., Ahtiainen J.P., Horttanainen M., Pollanen E., Makela K.A., Kainulainen H., Hakkinen K., Nyman K. (2013). Are skeletal muscle FNDC5 gene expression and irisin release regulated by exercise and related to health?. J. Physiol..

[21] Terada S., Goto M., Kato M., Kawanaka K., Shimokawa T., Tabata I. (2002). Effects of low-intensity prolonged exercise on PGC-1 mRNA expression in rat epitrochlearis muscle. Biochem. Biophys. Res. Commun..

[22] Fujimoto E., Yamaguchi W., Terada S., Higuchi M., Tabata I. (2011). Change in PGC-1alpha expression in rat skeletal muscle after low-intensity prolonged swimming exercise. J. Physiol. Anthropol..

[23] Roca-Rivada A., Castelao C., Senin L.L., Landrove M.O., Baltar J., Belen Crujeiras A., Seoane L.M., Casanueva F.F., Pardo M. (2013). FNDC5/irisin is not only a myokine but also an adipokine. PLoS ONE.

[24] Buscemi S., Corleo D., Buscemi C., Giordano C. (2018). Does iris(in) bring bad news or good news?. Eat Weight Disord..

[25] Anastasilakis A.D., Polyzos S.A., Saridakis Z.G., Kynigopoulos G., Skouvaklidou E.C., Molyvas D., Vasiloglou M.F., Apostolou A., Karagiozoglou-Lampoudi T., Siopi A. (2014). Circulating irisin in healthy, young individuals: Day-night rhythm, effects of food intake and exercise, and associations with gender, physical activity, diet, and body composition. J. Clin. Endocrinol. Metab..

[26] Besse-Patin A., Montastier E., Vinel C., Castan-Laurell I., Louche K., Dray C., Daviaud D., Mir L., Marques M.A., Thalamas C. (2014). Effect of endurance training on skeletal muscle myokine expression in obese men: Identification of apelin as a novel myokine. Int. J. Obes. Lond..

[27] Kraemer R.R., Shockett P., Webb N.D., Shah U., Castracane V.D. (2014). A transient elevated irisin blood concentration in response to prolonged, moderate aerobic exercise in young men and women. Horm. Metab. Res..

[28] Cousin B., Cinti S., Morroni M., Raimbault S., Ricquier D., Penicaud L., Casteilla L. (1992). Occurrence of brown adipocytes in rat white adipose tissue: Molecular and morphological characterization. J. Cell Sci..

[29] Lee P., Linderman J.D., Smith S., Brychta R.J., Wang J., Idelson C., Perron R.M., Werner C.D., Phan G.Q., Kammula U.S. (2014). Irisin and FGF21 are cold-induced endocrine activators of brown fat function in humans. Cell Metab..

[30] Terada S., Tabata I. (2004). Effects of acute bouts of running and swimming exercise on PGC-1alpha protein expression in rat epitrochlearis and soleus muscle. Am. J. Physiol. Endocrinol. Metab..

[31] Barreto T.O., Cleto L.S., Gioda C.R., Silva R.S., Campi-Azevedo A.C., de Sousa-Franco J., de Magalhaes J.C., Penaforte C.L., Pinto K.M., Cruz Jdos S. (2012). Swim training does not protect mice from skeletal muscle oxidative damage following a maximum exercise test. Eur. J. Appl. Physiol..

[32] Trevellin E., Scorzeto M., Olivieri M., Granzotto M., Valerio A., Tedesco L., Fabris R., Serra R., Quarta M., Reggiani C. (2014). Exercise training induces mitochondrial biogenesis and glucose uptake in subcutaneous adipose tissue through eNOS-dependent mechanisms. Diabetes.

